# Macrocystic Lymphangioma of the Chest Wall: A Rare Localization

**DOI:** 10.7759/cureus.102957

**Published:** 2026-02-04

**Authors:** Larbi Benradi, Mohamed Belahcen

**Affiliations:** 1 Pediatric Surgery, Mohammed VI University Hospital of Oujda, Oujda, MAR; 2 Faculty of Medicine, Mohammed First University, Oujda, MAR

**Keywords:** chest wall, lymphangioma, macrocystic, soft tissue, surgery, swelling

## Abstract

Lymphangioma (LA) is a congenital benign malformation due to lymphatic vessel proliferation, resulting from the lack of communication between the venous system and the primitive lymphatic sacs. They are essentially enlarged lymph nodes with chylous or serous content covered by the endothelium. The most common localizations are the neck, followed by the axilla. Cystic lymphangiomas can be classified into two types: macrocystic, which is the most common, and microcystic, which is less frequent.

In this article, we discuss the findings of a macrocystic lymphangioma of the right lateral chest wall in a five-year-old girl successfully treated in our department of pediatric surgery. The follow-up did not show any sign of recurrence after 12 months.

## Introduction

Lymphangiomas (LAs) are developmental abnormalities of the lymphatic system that most commonly occur in infants [1]. They are generally located in the neck, axilla, and other sites such as the mediastinum, retroperitoneum, and pelvis. LAs are rarely located in the chest wall [2]. LAs are generally divided into macro and microcystic forms. Based on histological features, three subtypes are identified: the cystic and the capillary LA, which are macrocystic and the most common, and the corpus LA, which is microcystic and represents the rare entity [3].

We present a case of a five-year-old girl presenting with a macroscopic LA of the right lateral chest wall.

## Case presentation

**Figure 1:**
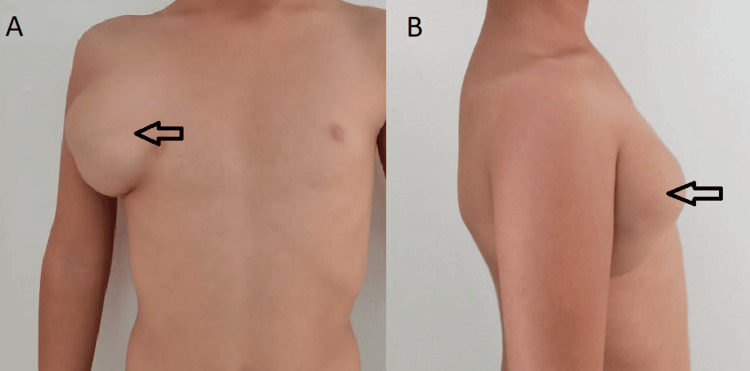
(A, B) A right chest wall swelling in a five-year-old girl, with no extension to the upper limb (arrow).

A five-year-old child with no notable pathological history was followed in our department of pediatric surgery for a congenital subcutaneous swelling of the chest wall. The mass gradually increased in size. The patient had no pathological history. Clinical examination found an apyretic child in good general condition with a poorly limited mass of the right chest wall, measuring 18 cm, renitent, and mobile on palpation, with a positive transillumination (Figure 1).

**Figure 2:**
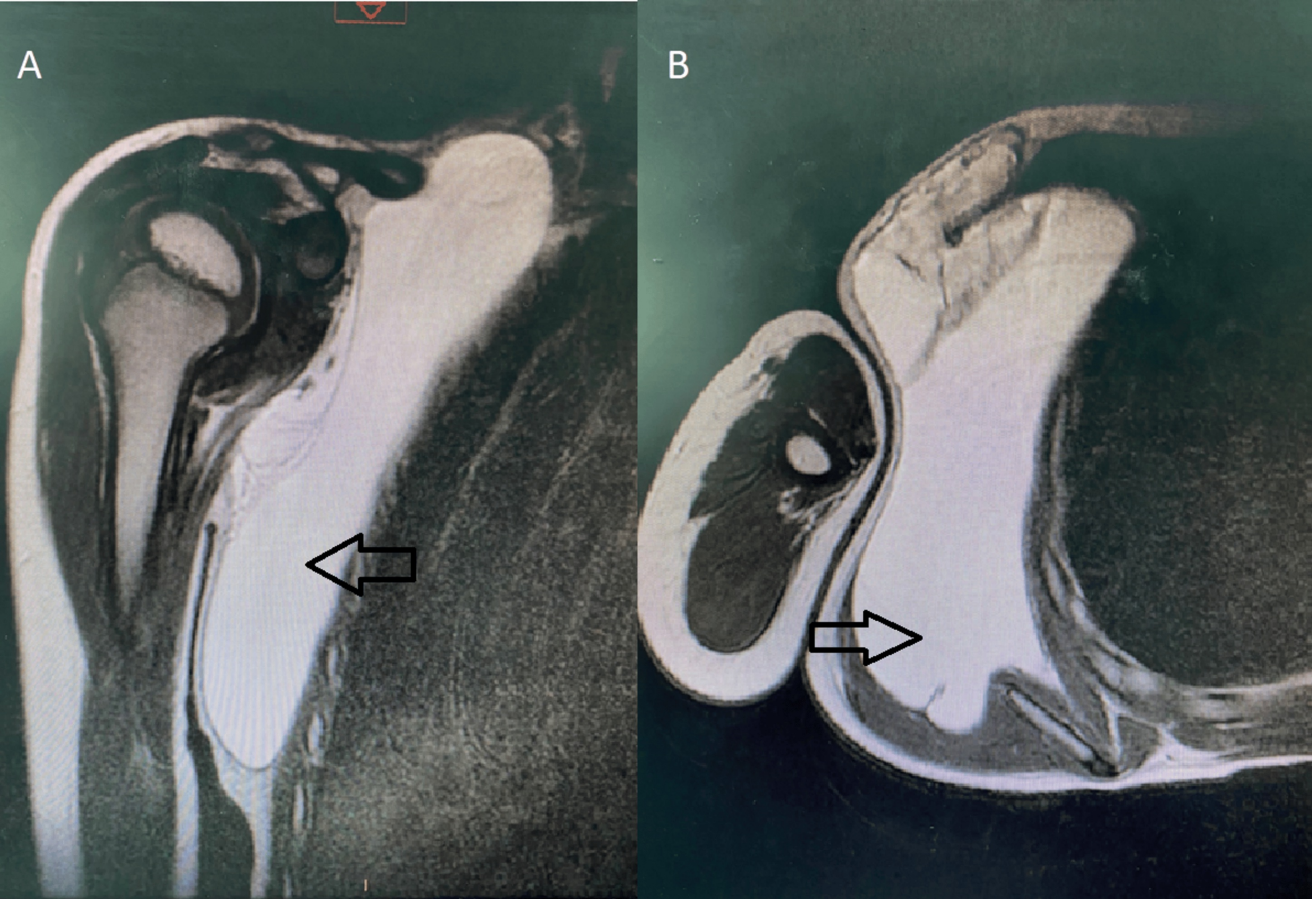
(A) Coronal magnetic resonance imaging showing a superficial swelling (arrow) with a fluid signal content. (B) Transverse magnetic resonance imaging slice revealing cystic swelling (arrow) of the right chest wall with regular seams on hypersignal T2.

Additionally, an ultrasound was performed and showed a cystic swelling of subcutaneous tissue of the right chest wall, well-limited, and measuring 16 x 9 cm with no vascularization on Doppler. Magnetic resonance imaging (MRI) revealed a cystic mass with fluid content and regular seams, measuring 154 x 82 mm, on hyposignal T1 and hypersignal T2, evocating macrocystic lymphangioma (Figure 2). All serum parameters were normal.

**Figure 3:**
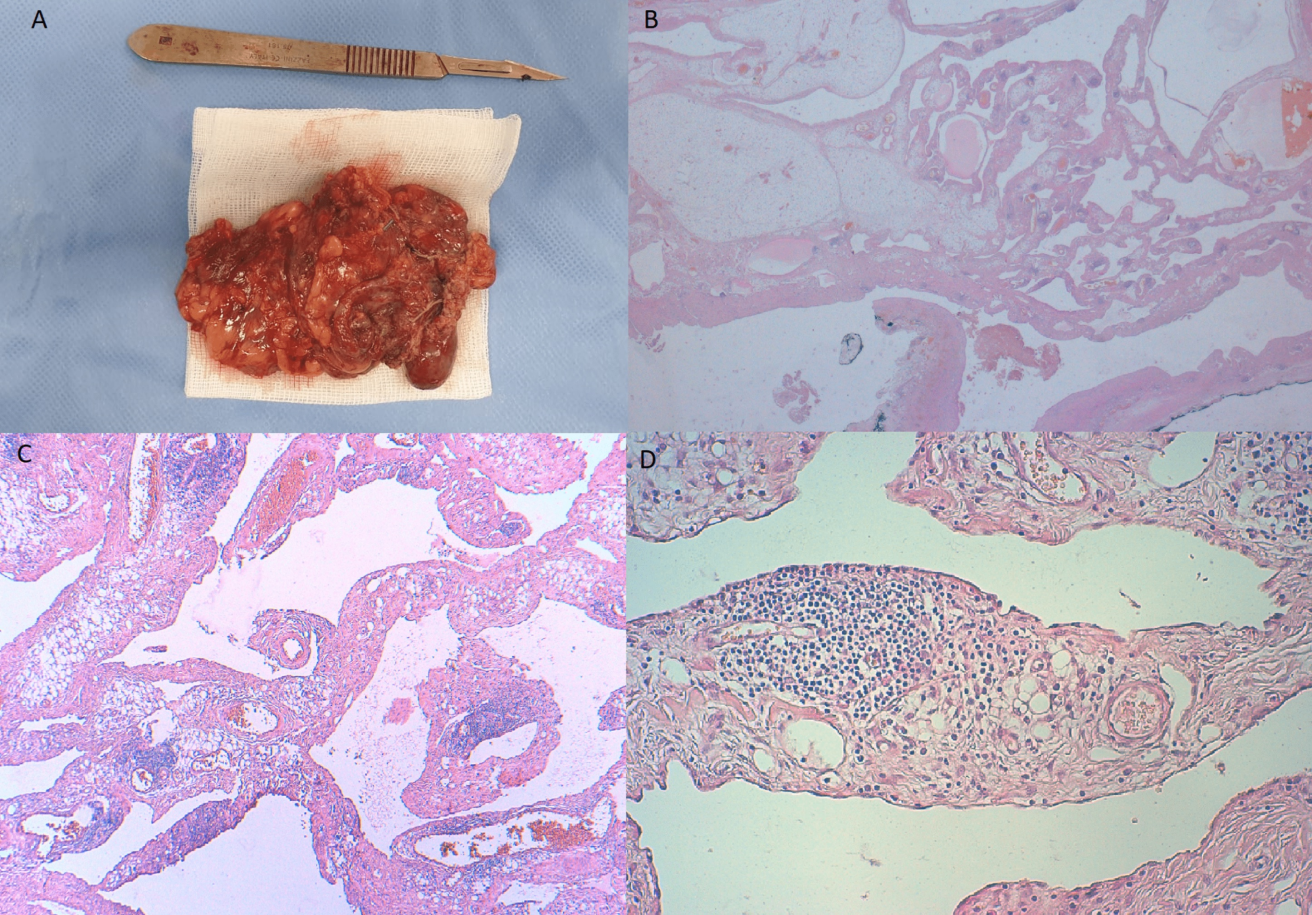
(A) Photograph of the resection specimen of a multilocular cystic mass. (B) Low-power view showing several irregular cysts, focally filled with eosinophilic fluid (x4 magnification). (C) Lymphatic channels of variable sizes, separated by loose connective tissue stroma containing numerous lymphoid aggregates (x10 magnification). (D) High-power view showing lymphatic spaces lined by flattened endothelium, with a lymphoid aggregate occupying the wall of the cysts (x20 magnification).

The patient was initially treated by five sessions of sclerotherapy using bleomycin, with a marked decrease in the size and change in texture of the mass, becoming solid and very well limited. In the second step, the patient benefited from a surgical treatment consisting of a complete resection by a thoracic approach. The postoperative care was normal with no hemorrhagic or infectious complications. The diagnosis of macrocystic lymphangioma was made based on the histological appearance (Figure 3).

After one year of follow-up, the patient was asymptomatic without any evidence of recurrence.

## Discussion

The incidence of cystic lymphangioma (CL) is about one in 12,000 births, and it represents 5-6% of all pediatric neoplasms [4]. More than half of CL is present at birth, and 90% appear during the first 24 months of life [2]. Also, the mean age of diagnosis is three years of life [5]. Thoracic CL generally occurs in the lung and mediastinum [6]. The chest wall as a localization is rarely noted and represents only 1% [6]. Here, we present the case of a right chest wall CL in a five-year-old girl with nonspecific clinical manifestations.

Clinically, CL presents as a large soft tissue cystic swelling with positive transillumination, allowing differentiation between cystic and solid tumors, as in our case [2]. However, transillumination is not feasible in some cases of CL after trauma, spontaneous bleeding, or complicated by infection [6]. On ultrasound, CL appears as a multicystic fluid mass with a regular shape and clear borders [3]. Doppler ultrasonography is useful to detect any vascular component. Furthermore, MRI allows a better analysis of the mass and the expansion to neighboring organs, especially large vessels, airways, and bone involvement [7]. In our case, MRI was chosen to better characterize the mass, to study its extension to the main deep vessels, and to assess possible extension into the axillary region, which allowed the surgical procedure to be properly planned.

Complete surgical resection is the basis of treatment, which is not always achievable [8]. Also, sclerotherapy using bleomycin, dextrose, or tetracycline as a component can be initially used to reduce the volume of the tumor, especially if there is close contact with nerve and/or vascular structures [9]. Nevertheless, skin erythema, local swelling, induration, slight tenderness, fever, and infection are reported as the most frequent sclerotherapy complications [10]. Additionally, recurrence after sclerotherapy as the first treatment choice for similar cases of CL in children can be encountered [11]. Bhatnagar et al. reported a case series of 15 patients with uncommon localization of CL treated with bleomycin sclerotherapy. A complete resolution was seen in 13 patients, and a partial resolution was observed in the two remaining cases, where a subsequent surgical excision was required [12].

Lymphangiectasis, other cysts, and soft subcutaneous masses might mimic a CL and represent the main differential diagnosis [13,14]. The final diagnosis of macrocystic lymphangioma is based on pathological analysis of the mass after surgical excision. Indeed, observation of the sectioned tissues reveals multiple lumens with no red cells, except for blood vessels corresponding to the cystic character of this tumor [15]. In addition, lymphatic vessel endothelial receptor-1 and D2-40 are considered the specific markers in immunohistochemistry [16].

## Conclusions

Lymphangioma of the chest wall is rarely seen in the pediatric population. Clinical assessment, especially MRI, is mandatory to characterize the lesion and to plan its treatment strategy. Surgery remains the basis of treatment, and sclerotherapy shows encouraging results, especially for uncommon and challenging cases.
